# Spinal cord leptomeningeal myelomatosis

**DOI:** 10.1016/j.htct.2025.103745

**Published:** 2025-03-06

**Authors:** Gustavo Kazuo Silva Yamada, Guilherme Duffles, Carmino Antonio de Souza, Fabiano Reis

**Affiliations:** a Faculdade de Ciências Médicas, Universidade Estadual de Campinas (UNICAMP), Campinas, SP, Brazil; b Universidade de Campinas (UNICAMP), Campinas, SP, Brazil

A 62-year-old man was admitted for investigation of a 3-month history of progressive lower back pain with hypoesthesia. He had been diagnosed with multiple myeloma 5 years before, treated with four cycles of CyBorD (cyclophosphamide, bortezomib (Bortezomib), dexamethasone) and pamidronate, followed by hematopoietic autologous stem-cell transplantation (conditioned with 200 mg/m² of melphalan) and maintenance chemotherapy with two cycles of CyBorD and isolated bortezomib (Bortezomib). In a regular medical follow-up, he had a very good partial response before admission. An examination showed paresthesia and hypoesthesia of lower limbs. Seric hemoglobin was 17.6 g/dL (normal reference [NR]: 14–18 g/dL), leukocytes of 8.23 x 10³/µL (NR: 4.0–10.0 x 10³/µL) subdivided in 5.61 x 10³/µL segmented neutrophils, 1.62 x 10³/µL lymphocytes, 0.73 x 10³/µL monocytes, 0.17 x 10³/µL eosinophils and 0.04 x 10³/µL basophils, without blasts, plasmocytes and other atypical cells. Magnetic resonance imaging (MRI) findings are shown in Figures 1 and 2. The imaging findings were consistent with leptomeningeal neoplasic infiltration, a condition called meningeosis myelomatosis,^1^, ^2^, ^3^ as a recurrence of the multiple myeloma. A cerebrospinal fluid (CSF) analysis was performed, which demonstrated plasmocytes with atypical morphology: increased volume, loose chromatin and evident nucleoli, that in a differential count was consistent with clonal plasmocytes.^3^, ^4^ Meningeosis myelomatosis is a rare but an important differential diagnosis to consider in patients with new neurological symptoms after multiple myeloma treatment. MRI is essential to evaluate the patients,^1^, ^5^ with a CSF analysis being the gold standard for confirming the diagnosis.^1^, ^4^

**Figure 1:**
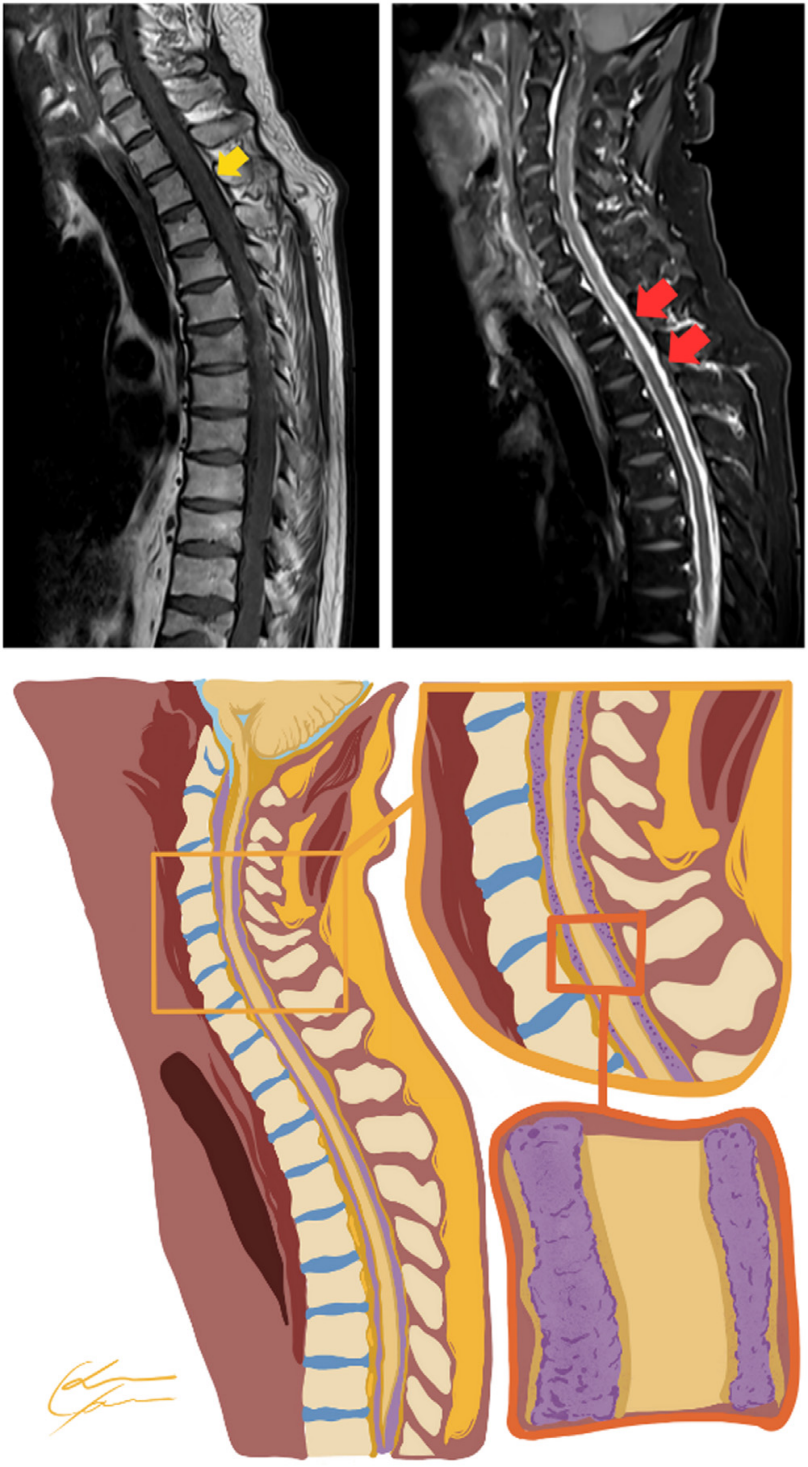
Sagittal T1 before (top left) and after contrast (top right). Sequences with diffuse and thick leptomeningeal enhancement involving the spinal cord. At the bottom, a graphic representation of the magnetic resonance imaging (MRI) findings with infiltration of the leptomeninges around the spinal cord.

**Figure 2:**
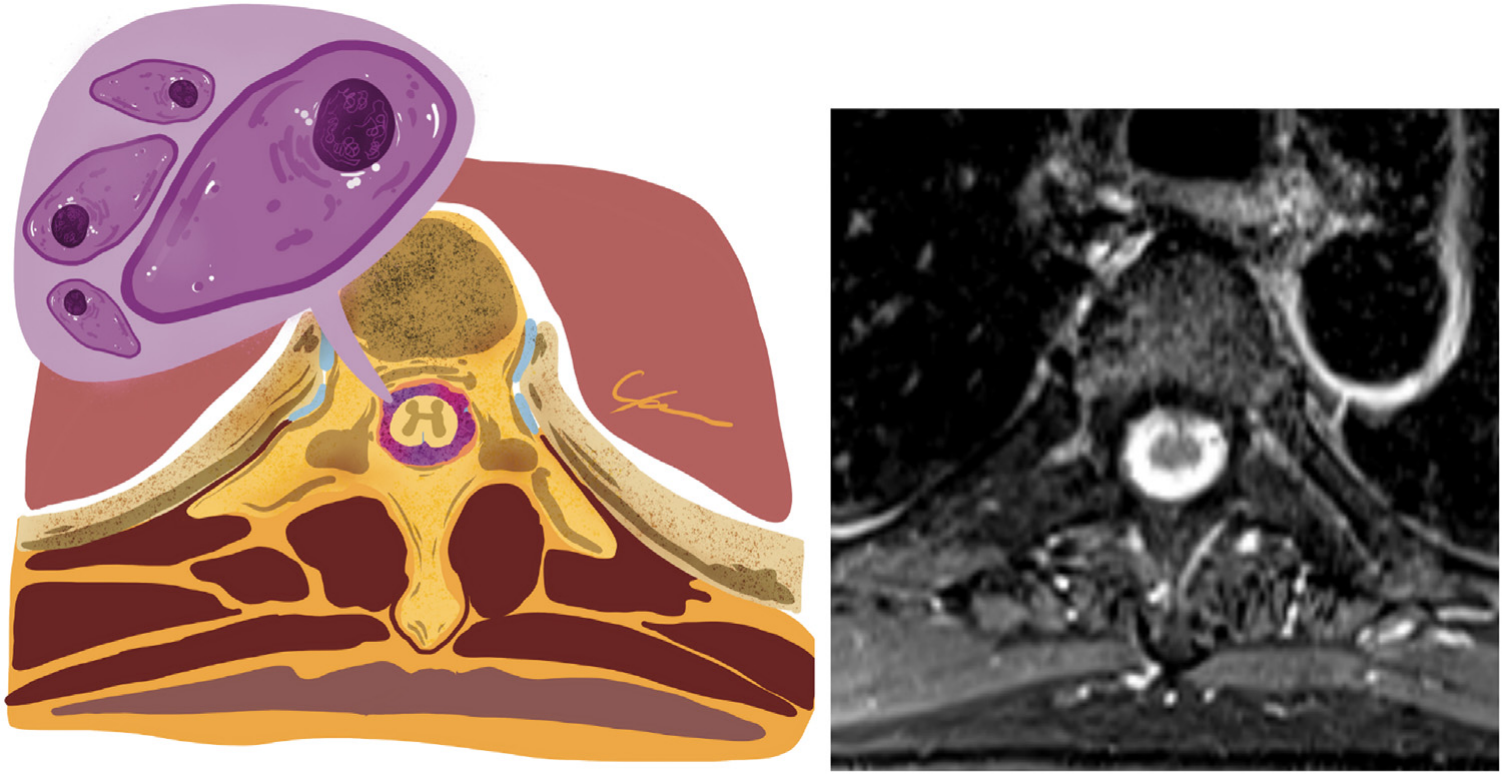
Graphic representation (left) from the Axial T1 after contrast (right) magnetic resonance acquired in the same patient. The representation illustrates the leptomeningeal neoplasic infiltration enhanced by contrast in T1 sequences that was confirmed to be by plasmocytes with atypical morphology (enlarged cells, loose chromatin and evident nucleoli), a rare recurrence of multiple myeloma.

## Conflicts of interest

The authors declare no conflicts of interest.
